# The Incidence of Pediatric and Adolescent Concussion in Action Sports: A Systematic Review and Meta-Analysis

**DOI:** 10.3390/ijerph17238728

**Published:** 2020-11-24

**Authors:** Francesco Feletti, Matteo Bonato

**Affiliations:** 1 Department of Diagnostic Imaging, Ausl della Romagna, S. Maria delle Croci Hospital, 48121 Ravenna, Italy; 2 IRCCS Istituto Ortopedico Galeazzi, Via Riccardo Galeazzi 4, 20161 Milan, Italy; matteo.bonato@grupposandonato.it

**Keywords:** action sports, concussion, injury, diagnosis, orthopedics

## Abstract

Background: This was a systematic review and meta-analysis of the incidence of concussion risk in youth athletes involved in action sports (AS). Methods: A search of PubMed and Web of Science (from January 1980 to August 2020). Titles, abstracts, and full text were screened according to predefined inclusion criteria to find relevant studies. Moreover, the methodological quality of the studies selected was assessed. Results: Nineteen of 1.619 studies were included in the systematic review and 14 in the meta-analysis. Motocross, sailing and snowboarding presented the highest incidence rates per 1000 athlete exposure at 39.22, 3.73 and 2.77 respectively, whereas alpine skiing had the lowest incidence rates resulting in 0.30. Overall risk of concussion was estimated at 0.33 (CI: 0.22, 0.45). Regarding the methodological quality, we have to report that 26.3% of the studies reported the definition of concussion while 36.8% presented age and gender-specific incidence rates. The mechanism of injury and follow up were reported only in one study. Conclusions: There are significant differences in the rates of incident youth concussion across AS. Despite some limitations, the data from this research can serve as the current sport-specific baseline risk of concussion among youth athletes who practice action sports.

## 1. Introduction

In the last two decades, non-traditional sports activities characterized by elements such as speed, height, and exposure to natural forces knew a rapid increase in global participation. They are generally referred to as action sports (AS), with the terms adventure sports or extreme sports that could be used as interchangeable synonyms [1,2,3,4]. In particular, the perception of an inherent risk has been highlighted by the mainstream media as an essential aspect of these activities, and often even considered their main distinguishing feature [2]. However, this preconception is currently not supported by enough evidence describing the real incidence of injuries in these sports [3,5]. Instead, unlike traditional sports, AS are the occasion of mastery and perfection in a challenging environment. Moreover, aesthetic criteria such as the personality of “style”, innovation and originality play a crucial role when assessing performance in AS [6]. As a part of this growth AS have proven to be attractive to young people [7]. To this regard, the inclusion of youth AS such as sport climbing, skateboarding, surfing, snowboarding, kitesurfing in the Youth Olympic Programs is driving the popularity of these activities among children and adolescents [4,8]. For example, in the United States in 2019 among youth aged 6 to 17, there were 12.7 million of participants in off-road mountain biking, 3.7 million in skateboarding, 3.1 million in snowboarding, 2.8 million in skiing and 808,000 in sailing [9].

Youth participation in AS, on the one hand, may result in a wide range of physical health benefits, including reduction of body fat, enhancement of bone health and improvement of the physical fitness, the cardiovascular and metabolic disease risk profiles. Furthermore, AS participation may promote social development, resilience, self-worth and self-control [7] Among young participants in AS augmented informal collaborative developmental processes have been observed [7]. Finally, AS might be more effective at building communities, enhancing social development and generating environmental awareness than traditional sports [7].

On the other hand, however, AS may involve risk of injuries [10]. Specifically, adolescents and children who practice ASs may be particularly prone to concussion due to more significant head to body ratio, immature coordination, reduced skills and lower awareness of risk, especially in the presence of peer [4,11]. Children’s brain have a massive growth spurt when they are very young. By the time they are six, their brains are already about 90–95% of adult size, but the brain still needs a lot of remodeling before it can function as an adult brain. Brain remodeling happens intensively during adolescence, some changes happens intensively before puberty, and some continue long after. Functional brain change in particular, depends on age, experience and hormonal changes during puberty [12]. During this process, AS have proven to be attractive to young people [7]. Specifically, during adolescent brain development, the modifications in structural morphology, structural connections, functional connections, and task-related activity may play a role in explaining individual patterns of risk-taking behavior. For example, as children grow up, structural and functional connectivity between the striatum and dorsal medial and lateral prefrontal cortex increases, leading to more future-oriented, less impulsive choice. Intriguingly, neural activity in both the ventral striatum and ventromedial prefrontal cortex is heightened in mid-adolescence relative to childhood and adulthood. Therefore, during maturation of ventral striatum-PFC connectivity, there is possibly enhanced sensitivity of this reward-seeking and valuing network, which is associated with a peak in certain types of risk-taking behavior. Possibly, a temporarily less inhibited system allows adolescents to explore their environment, seek out new relations and differentially value information that they receive [13]. Individual genetic predisposition as well as brain chemicals processes may drive the need to search for AS, activities that are thrilling, uncertain, novel, ambiguous and unpredictable [7].

The data from a review of the injuries sustained over 12 years, during the practice of seven popular AS featured at the X-Games (data from the National Electronic Injury Surveillance System: NEISS) confirm that teens and adolescents accounted for the highest percentage of injuries and concussion represented the 3.4% (*n* = 140,650) [5]. To this regard, it has been demonstrated that individuals sustaining mild traumatic brain injuries often report a constellation of physical, cognitive, emotional and behavioral symptoms referred as post-concussion symptoms. The most commonly reported post-concussion symptoms are headache, dizziness, decreased concentration, memory problems, irritability, fatigue, visual disturbances, and sensitivity to noise, judgment problems, depression and anxiety. Although, these post-concussion symptoms often resolve within one months, in some individuals can persists from months to year following injury and may even be permanent and cause disability [14]. Developmentally younger brains may present an increased vulnerability to a concussion, as well as longer recovery and different physiological response after this specific injury [4,11]. Moreover, a concussion may adversely affect future health due to post-concussion syndrome and result in re-injury in case of returning to AS without complete recovery [10]. It is valuable for all healthcare providers to be aware of these risks so they can adequately educate families, coaches, the athletes themselves and the institutions to adopt all the necessary measures to reduce the risk of concussions. At the same time, the awareness of the incidence of concussion among young participants in ASs may help physicians to develop specific strategies and guidelines for the treatment and rehabilitation of young athletes.

With these premises, the present study aims to examine the epidemiology of concussion in action sports, as they have been defined, according to Immonen et al. [6].

## 2. Methods

### 2.1. Study Design

A systematic review and meta-analysis of medical literature were performed using the standardized guidelines for systematic reviews and meta-analyses proposed by Harris et al. [15], following the guidelines for meta-analysis of observational studies in epidemiology (MOOSE) [16], and following the checklist for the Preferred Reporting Items for Systematic Reviews and Meta-Analyses 2015 (PRISMA) [17]. A protocol addressing the precise identification of Participants, Interventions, Comparisons/Controls, Outcomes, and Study Design (PICOS criteria) was preliminarily submitted to the International Prospective Register of Systematic Reviews (PROSPERO) [18] and registered with number CRD 42020204513.

### 2.2. Information Sources

One researcher (FF) independently searched the PubMed and Web of Science electronic databases. The search covered all the available literature from January 1980 to August 2020, with the date of the last search being August 2020. Any articles identified by the search that were deemed relevant (based on title and abstract) were sent to another researcher (MB) for full-text eligibility assessment. Both researchers (FF and MB) double-checked the included papers from this assessment and modified the eligibility criteria according to the scope of the systematic review. Hand searching of reference list from bibliographies of included studies was also used to identify articles.

### 2.3. Eligibility Criteria

To be included, studies had to: (i) be written in English; (ii) have been published in a peer-reviewed journal; (iii) include a series of at least ten subjects; (iv) aim to study the incidence of injuries in youth athletes involved in AS. Conference abstracts and theses were excluded due to the difficulty in obtaining full methods and complete data sets and, therefore in assessing the risk of bias and data analysis.

### 2.4. Search Strategy

A Population Intervention Comparator Outcome (PICO) strategy was used to build search criteria for electronic databases on four comprehensive search themes. In particular, Theme 1 included terms to identify relevant outcomes: “concussion” or “brain concussion” or “brain injuries” or “mild traumatic brain injury” or “head injury”. To identify relevant sports, as Theme 2, we included the terms: “High-risk sport” or “Alternative sport” or “Lifestyle sport” or “Action sport” or “Extreme sport” or “Action sport” or “Adventure sport” or “Sports” or “Sport” or “Athlete”. Theme 3 included words to identify appropriate populations: “Adolescent” or “Youth” or “Child” or “School” or “Students”. To identify suitable study designs, as Theme 4, we added the terms: “Cross-Sectional Studies” or “Prevalence” or “Cohort Studies” or “Follow-up Studies” or “Incidence”. These four themes were combined using the Boolean operator “and”. Terms were searched as text words (or abstract/title words), and these four themes were combined using the Boolean operator ‘and’ to complete our search strategy (Supplementary material). The search strategy was mapped to an appropriate subject heading for each of the databases used for this systematic review and meta-analysis. No filters or limitations were imposed during the search.

### 2.5. Study Selection

Manual methods removed duplicate references. The two authors (FF and MB) independently screened titles and abstracts to determine the initial eligibility. Blinding of authors was used to reduce bias during this process. Finally, the authors reviewed the full-text for inclusion based on the eligibility criteria. The articles for which there was indecision about eligibility were also full-text reviewed. Full-text articles were retained if they met the inclusion criteria of study design: relevant sports (mountain-biking, motocross, paragliding, hang gliding, speed riding, parachuting, skydiving, BASE jumping, sport climbing, mountaineering, parkour, skateboarding, windsurfing, sailing, kiteboarding, wakeboarding, surfing, skiing, snowboarding, snow kiting, in-line skating, winter sliding sports), relevant population (males and females ≤18 years), and outcome (concussion). Disagreement in eligibility decisions was resolved by consensus. Inter-rater agreement was measured with the κ statistic.

### 2.6. Data Extraction and Analyses

One author (FF) using a standardized form, that was pilot tested on ten randomly selected studies and refined accordingly, completed data extraction. The other author (MB) then merged the data, and any discrepancies in the extracted data were resolved through discussion. Among the articles, we carefully selected those providing a concussion incidence rate or report both the crude number of concussion and an adequate denominator (e.g., days of athlete exposure, number of participants, hours of sport exposure, population served by a trauma centre). We chose not to enforce a strict definition of concussion for this review, since the definition of concussion has been subject to change over the years. Moreover, any direct shocks to the neck face or other body areas that transmit acceleration to the head are includes in the definition of concussion provided by the International Conference on Concussion in Sports [19]. Therefore, the imposition of a restricted definition of concussion (e.g., resulting from blunt trauma or direct trauma to the head) may alter the selection of relevant studies. Our primary goal was to elucidate the incidence of concussion in children and adolescents ≤18 years of age engaging in action sporting activities and assessing how the risk diversified across specific disciplines.

For this reason, we chose to eliminate articles that reported prevalent, rather than incident cases of concussion, as well as series of injuries missing any population denominator, and studies that included chronic traumatic brain injury. We also made attempts to contact authors of papers where data remained unclear. We manually extracted the following data: number of participants, age range, number of concussions, the denominator (person-time at risk or number of athletes exposed) and whenever provided the incidence rate of concussion. Similarly, to what already done in more traditional sports, we used an adapted version of The Newcastle Ottawa Scale for the evaluation of study quality of observational studies [20]. Specifically, we assessed how the study cohorts were chosen and covered, whether study outcomes were stratified by essential factors in their analysis (age and sex). We also considered how the outcome of interest (concussion) was diagnosed and measured (data relative to follow-up, the definition of concussion, who reported the data, mechanism of injury reported, previous history of concussion reported, dynamics of the traumatic event, whether the data were relative to competition or simple practice). Specifically, we addressed the incidence of concussion in the following sports: mountain biking, motocross, paragliding, hang-gliding, skydiving, base jumping, sport climbing, mountaineering, parkour, skateboarding, in-line skating, kitesurfing, wakeboarding, sailing, windsurfing, surfing, skiing, snowboarding, and many of their subdisciplines. We excluded from our analysis team and contact-sports (e.g., ice-hockey, box, etc.).

### 2.7. Statistical Analysis

The incidence of concussion was reported in diverse ways, the two most common denominators being days of athletic exposure (DAEs) and player-year (PY). Since most studies report incidence in terms of DAEs, we planned to pool those studies reporting DAEs and whenever possible, to convey back the data to this unit of measurement. In an attempt to compare the incidence of concussion in dinghy sailing (where the denominator was expressed in terms of hours of exposure), we converted player hours to DAEs for this sport. To do this, we assumed the length of a session in Europe or Laser dinghy sailing classes being 2 h and 30′. This estimation was based on the personal racing experience of more than ten years in the Laser class of one of the authors (FF).

Meta-analysis was limited to studies reporting DAEs and was conducted adopting the DerSimonian and Laird random-effects model. We calculated a pooled estimation of the incidence rates relative to single or groups of sports whenever available data from two or more unique studies. We also obtained an estimation of the overall incidence of concussion across the action sports discussed in the meta-analysis. To visualise the incidence rates and to correspond 95% CIs within and across sports, we generated a forest plot for all sports. In order to measure the heterogeneity, we calculated both Q and I^2^ statistics. We expected significant heterogeneity both within and across sports. All analyses were performed using the software JASP 0.13.1 (The University of Amsterdam, Amsterdam, The Nederlands) and a result *p* < 0.05 was considered statistically significant.

## 3. Results

**Figure 1:**
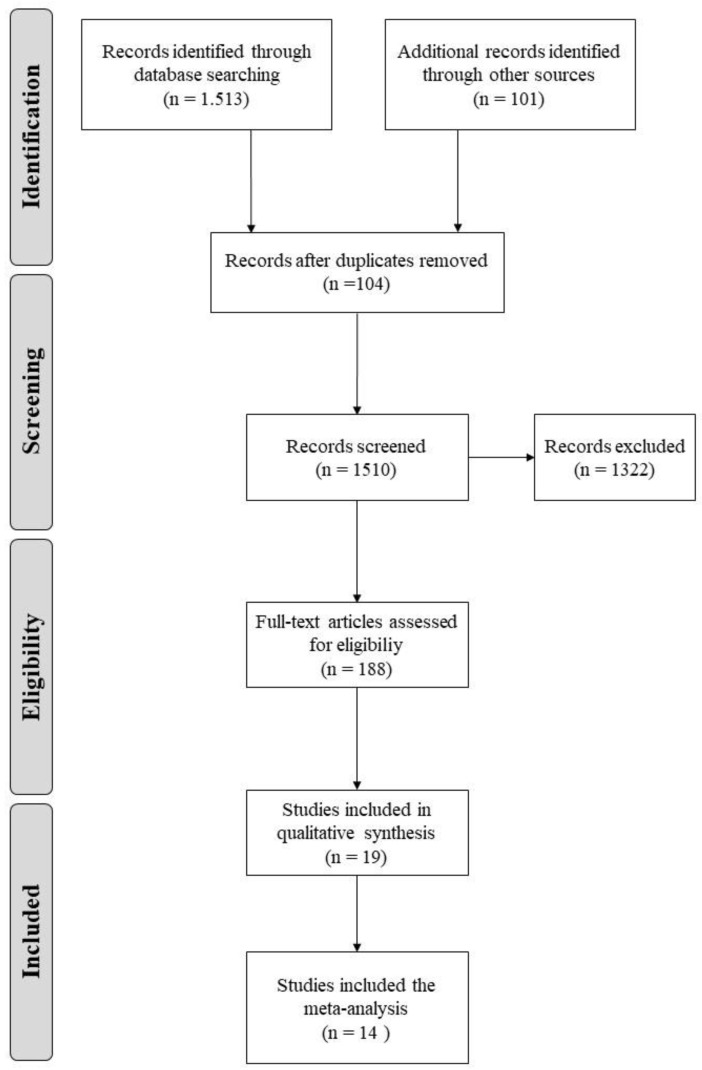
Flowchart of the selection process for inclusion of articles in the systematic review and meta-analysis.

Figure 1 reports the PRISMA flowchart representing the step-by-step process of identification and application of exclusion criteria leading to the final number of studies included in the systematic review and meta-analysis.

Our initial search identified 1614 unique citations. At this stage, 104 articles were duplicates and therefore removed. Of the remained 1510 records, 1322 were excluded, with 188 records that met criteria for full-text review with finally 19 studies included in the qualitative analysis and 14 in the meta-analysis. Characteristics of the 19 included studies are provided in Table 1.

The primary reason for exclusion of studies was lack of data about the relevant population (outdoor sports participants ≤18 years of age) and studies with participants of ≤18 years old, but which did not report the concussion incidence rate nor the combination of the crude number of concussion and an adequate denominator. The publication dates ranged from 1982 to 2020; the number of sports examined from each paper ranged between 1 and 5, and up to three disciplines (e.g., Bobsleigh, Luge and Skeleton) were grouped for each sport.

In total, seven sports were identified including alpine skiing (*n =* 10), freestyle skiing (cross, halfpipe, slopestyle, *n =* 2), snowboarding (halfpipe and slopestyle, *n =* 12), off-road motorcycle/motocross (*n =* 2), dinghy sailing (Europe and Laser classes, *n =* 1), sliding sports (bobsleigh, luge and skeleton, *n =* 2), skating sports (figure, short track and speed, *n =* 1). The papers discussed data related to six countries: Austria (*n =* 4), USA (*n =* 5), Canada (*n =* 6), Australia (*n =* 1), Norway (*n =* 2), and Sweden (*n =* 1). Ten studies did not specify an age range (range 4.0–18.0), but seven defined the upper level (≤18), while three provided the mean age (range: 14.7–17.0). The range of the young population involved spanned between 21 and 720,066 participants, with two papers not reporting the total number of participants included. Ten studies reported the proportion of male population ranging from 50.0% to 89.5%. Apart from two studies which reported the incidence of concussion in terms of mean days between injuries (MDBI) all the other studies reported the number of concussions (range 1–39,364).

In contrast, a denominator was reported in all but five of the remaining papers. In these series, we recalculated the denominator using the number of concussions reported and the reported incidence of concussion. Furthermore, the studies differed for the denominator used to calculate the incidence of concussion. Three used player-years as the denominator, one reported the general population served by the involved emergency medical services, while all the remaining adopted the Days of Athletic Exposure (DAE).

There were some notable differences in the quality of the studies (Table 2). Concerning cohort selection, all 19 studies appear to have a representative exposed cohort.

Results of reported quality assessment are presented in Table 3. For the quality of outcome measures, four of the 19 studies (21%) reported a definition of concussion. Thirteen studies (76.4%) provided information about who reported the number of concussions: a physician in six studies, a member of the medical staff in three cases, a physician or an allied healthcare provider in two, and a ski patroller in three cases. None of the studies reported the mechanism of injury, the duration of follow-up, the history of previous concussions. Only five studies (29.4%) provided adequate information and stratification by essential factors such as age or sex. At the same time, only eight studies (47%) reported the competitive or amateur nature of sports practice described. Due to the scarcity of these data, no attempts were made to quantify the effect of these different constituents of study quality on concussion rates. A stratification of concussion severity by age and experience level was reported by Sran et al. [32] in their study on alpine skiing and snowboarding: with all severe concussions (35%; *n =* 6) being attributable to the older participants (age: 7–12) and more expert athletes (Ability Level: 3–4; scale range 1–4).

Finally, Gil et al. [21] reported a difference in the incidence between males and females partaking in skiing and snowboarding. Specifically, in skiing the incidence rate was 0.0315/PY for males and 0.0198/PY among female, while in snowboarding it was 0.0283/PY and 0.0101 respectively. In the series by Luo [39] among motocross participants suffering from concussive symptoms, 24% of the riders did continue to actively participate in their sport while the remaining only briefly suspended participation (mean 2.7 weeks; range 0–36 weeks), 78% (*n =* 52) sought treatment from a health care professional while 33% (*n =* 46) reported multiple episodes of concussion symptoms during the season.

**Figure 2:**
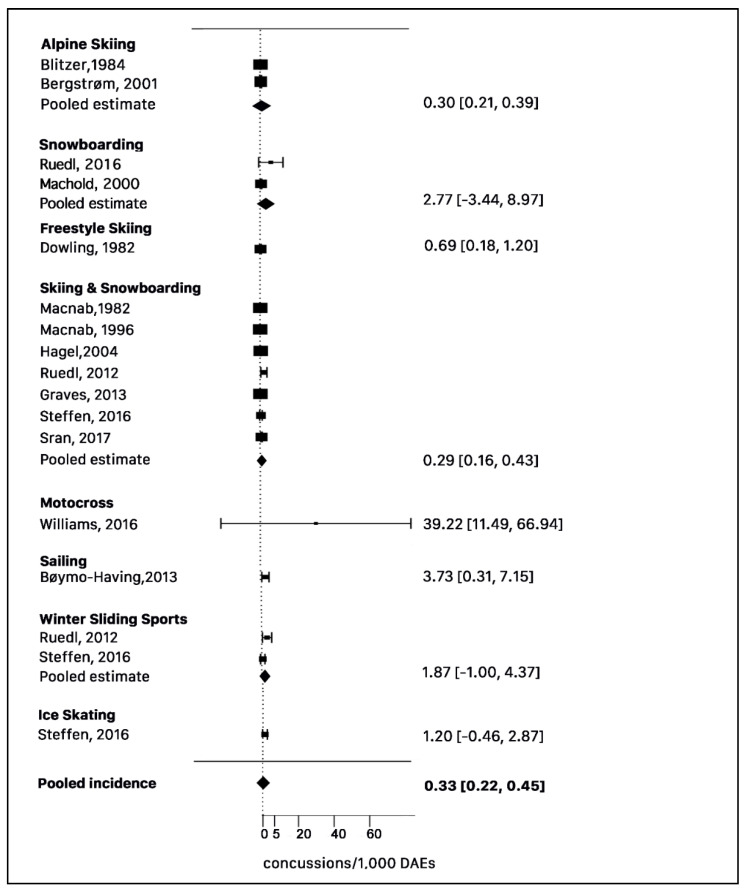
Forest plot of the sport-specific concussion incidence rates (random-effects model).

Apart from motocross, also for alpine skiing, sailing, curling and ice-skating, there was only one estimation of concussion incidence. We pooled the incidence rates of those sports with two or more unique studies, which adopted DAEs as the denominator. The pooled incidence of concussion per 1000 DAEs across eight sports in 13 studies using a random-effects model was 0.33(95% CI 0.22 to 0.45) (Figure 2). Significant heterogeneity in this pooled between-sport estimate (Q statistic: *p* < 0.001). In the meta-analysis, the incidence of concussion (per 1000 DAE) ranged between 0.30 (95% CI: 0.21 to 0.39) of skiing to 39.22 (95% CI: 11.49 to 66.94) of motocross. However, the latest data was based on a single study. Apart from motocross, sailing showed the highest incident rate per 1000 DAEs at 3.73 (95% CI: 0.31 to 7.15). Within the pooled data, significant heterogeneity as defined by the Q statistic (*p* < 0.001) was found among the seven studies which grouped with the data relative to skiing and snowboarding as well as among alpine skiing studies. On the contrary, heterogeneity was not significant among the studies relative to the winter sliding sports (*p* = 0.202) and snowboarding alone (*p* = 0.382). Only one study relative to slope skiing and snowboarding [22] reported the data relative to age differences between children (aged 4–12 years) and adolescents (aged 13–17 years) with an incidence rate of 0.16 (95% CI 0.12 to 0.29) and 0.31 (95% CI 0.28 to 0.34) respectively.

## 4. Discussion

The popularity of AS is rapidly increasing among children and adolescents. The fashionableness of AS among youths is favored by the progressive inclusion of AS in the Youth Olympic programs. As a result, the participation rates of young people in AS are increasing [4], and related concussions may be likely increasing as well.

Sharma et al. [5], in their study, including all age groups, reported a significant increase in the number of head and neck injuries in AS from 2000 to 2011. Specifically, they reported an incidence of concussion of 5.16 per 1000 person-year; a value ranging from 0.05 in mountain biking to 0.534 in snowboarding [6]. Seventy-four per cent of the recreation or sport-related concussion admitted to a paediatric level 1 trauma service were caused by high-velocity activities, among which were skiing, snowboarding, motocross, and skateboarding [40]. Again, in the paediatric population, concussion spans from 5.0 to 51.5% of all reported injuries in snowboarding, mountain biking and skiing [4], and represents 1.1–82.6% of the injuries reported in skateboarding [1,4]. Despite the existence of many series relative to the injuries reported to hospitals and trauma centers, in our review we found only a few studies providing an adequate denominator for the calculation of the true incidence of concussion among participants. The critical efforts profuse to the understanding of the descriptive epidemiology of injuries related to AS in general and youth participants in particular, there is a lack of quantitative data assessing injury incidence [1].

In the present meta-analysis, we estimated an overall incidence of concussion in children and adolescents aged ≤18 years involved in outdoor sports of 0.33/1000 DAEs. We speculate that DAEs represents an excellent way to represent the actual exposure to the risk related to the practice of AS. While in traditional sports, the recommendation is to report the incidence of injuries in terms of cases per 1000 h of practice [41,42,43], this approach may result challenging in AS due to the typically intermittent nature of these activities [44,45]. Indeed, in AS the injury rate is often presented using very specific denominators, such as injuries/1000 skydives [46] or 1000 BASE-jumps [47]. Therefore, the use of DAEs as a denominator to assess the incidence of injuries in outdoor sports may have some crucial justifications: it is referred to the specific population of interest, it allows a standard span and comparable of time, and finally, it amalgamates active practice and intervals that may have different characteristics in different activities.

On the contrary, the alternatives encountered in the present review were unspecific and generic. Specifically, athlete exposure in terms of AY means that one participant was involved in outdoor sports practice regardless of the precise amount of time spent practicing. The use of the general population is even more generic since the practice of AS may differ between locations, and such a dominator include non-active population.

Our systematic review resulted in a limited number of series, with a complete lack of coverage of some famous sports, such as skateboarding or kite surfing. Causes may depend on the difficulties encountered in the scientific research in these sports often practiced alone or in small groups in remote places [48]. From our meta-analysis motocross resulted in a disproportionately high incidence of concussions compared to other AS. It is relevant that it is the only motorized AS because a higher risk of traumatic brain injury due to the engine thrust and equipment weight has already been reported among participants in AS motorized disciplines [49]. Concussions in young athletes may be underestimated because coaches and athletes themselves may poorly recognize them. In AS, this may be even truer, due to the low level of organization of these sports. Indeed most AS lacking structured organizations such as clubs or federations, which can organize data collection.

The present systematic review and meta-analysis may be relevant for several different knowledge users. Youth action sports participants, their coaches and families can become aware of the risk of suffering from a concussion in their specific sport. Parents are not satisfied by conjectures and want to know if AS are safe for their children [4,50]. While the general population may be aware of the concussion risk related to the practice of contact sports, the risk of sustaining a risk in sports such as sailing or motocross may be underestimated. Public health officials could use this information to address injury prevention strategies, especially in the organization of action sports events. Moreover, the awareness of the concussion risk in AS may improve concussion reporting and diagnosis, which is crucial because youth athlete is more susceptible to concussion and takes more time to recover [21]. The research about the protective effects of helmets against concussion is inconclusive. However, Luo et al. [39], reported that youth motocross riders that received professional help with helmet fitting had 41%decrease in reported symptoms of concussion (RR 0.59, 95% CI: 0.44–0.81). Other strategies such as specific guidelines and rules may be useful to reduce head injury incidence, but involve a delicate balance between safety, thrills, and any real danger, in order to preserve the exciting nature of AS [51]. Healthcare providers may use the data of the present study in guiding informed decision-making regarding appropriate medical care staffing and developing sport-specific management. In particular, with this systematic review and meta-analysis differences were observed in the injury rates and distribution between AS. Those epidemiologic data may be useful for steering future safety research, to allow participants and governing bodies to develop relevant sport-specific safety policies concerning training, event organization, protective clothing, equipment, and other safety systems.

A crucial limitation of the present study is the significant heterogeneity in the overall concussion estimate across the sports, which may depend on some different factors. First, it is the result of the differences between sports and their sub-disciplines; the different levels of acceleration involved the dynamics of injuries and differences in protective equipment used. Then it may depend on the different design of the studies, and the different sources of the data used as the denominator. We did not examine the sources of heterogeneity because of critical methodological issues in the reviewed studies, the limited number of studies available for each sport, and multiple sources of heterogeneity within and across sports. For example, the lack of stratification concerning sex may be a further source of heterogeneity. Indeed, gender may be an additional essential source of heterogeneity as females have a potentially greater risk of sustaining a concussion due to a reduced head and neck mass and a more significant angular acceleration of the head and neck [52]. However, in this review, the only study [21], which reported that gender-specific incidence showed a higher incidence of concussion among males. A possible reason is that in AS, males may be at higher risk of injuries because they tend to perform a wide selection of riskier maneuvers due to overconfidence and recklessness [1,3]. Another significant limitation is the lack of a definition of concussion in many studies. The diagnosis of concussion may be difficult as the symptoms may be subtle, and in many cases, a high level of suspicious is required for the diagnosis, thus resulting in underestimation of the number of concussion. It is also possible that some studies generically included head trauma or injuries, which fall outside the true definition of concussion. Furthermore, we did not address the influence of study quality on the results because many data were not reported in many studies, for example, the stratification in respect to age was not reported in most series, while critical data such as the previous experience of concussion, the follow up and the mechanism of injuries were generally not reported. Most studies failed to identify whether concussions occurred during competition or simple practice. Further cohort, prospective studies should focus on those AS for which the present review did not find reliable data about the incidence. Again, there is a need for more data about the follow up of concussed athletes, in order to understand better how concussion affects the sports practice and the life of AS youth participants. Finally, we did not specifically include longitudinal studies in the search strategy since this study design is not the standard method of research in the field of AS [4].

Future research should explore the dynamics of injuries, their differences across the various AS, as well as exploring the existence of sport-specific risk factors. More work is also required to translate data about concussion in education and awareness about risks preventive tools and diagnosis of concussion.

## 5. Conclusions

In conclusion, the present study may provide valuable insight into the incidence rates of concussion across eight AS among youths. The data may provide a baseline to further research on concussion in AS, which should be tailored to the specific disciplines. The data from this study could provide a baseline for further studies on concussion in AS that can highlight sport-specific traumatic dynamics, the risk factors involved and which equipment and guidelines can be useful in preventing and managing concussion in young people. Concerns related to susceptibility and concussion recovery in younger athletes make it necessary to focus research on young athletes. It is essential that parents, coaches, health professionals and athletes understand the specific risk level of concussion of each sport and are instructed in the detection and assessment of concussion, to allow the suspension of potentially risky activities and initiate a rehabilitation that allows the safe return to sports.

## Figures and Tables

**Table 1:** Study characteristics of the 19 articles included in the systematic review.

STUDY		Data Source	Number of Subjects	Age Range	Male Population (%)	Period of Observation
First Author, Year	Sport/s					
Gil et al. [21]	Alpine skiing	National Electronic Injury Surveillance System (NEISS) (USA)	Unreported	0–19	Unspecified	1 season (2014–15)
	Snowboarding	National Electronic Injury Surveillance System (NEISS) (USA)	Unreported	0–19	Unspecified	1 season (2014–15)
Graves et al. [22]	Alpine skiing& Snowboarding	National Electronic Injury Surveillance System (NEISS) (USA)	60,626	4–17	72.8	14 seasons (1996–1997/2009–2010)
Emery et al. [23]	Snowboarding	Random sample of classes from 24 Calgary and area high schools (Canada)	579 *	14–19	Unspecified	1 year (2003–2004)
Macnab et al. [24]	Alpine skiing and snowboarding	Skiers and snowboarders visiting a world class ski resort (Canada)	70	<13	Unspecified	1 season (1998–1999)
Macnab et al. [25]	Alpine skiing and snowboarding	Skiers and snowboarders visiting a world class ski resort (Canada)	720,066	0–17	Unspecified	1 season (1991–1992)
Williams et al. [26]	Motocross	Participants at annual motocross competition in North Central Florida (USA)	51	8–17	92.1	1 day competition/4 editions (2009–2012)
Blitzer et al. [27]	Downhill skiing	Skiers at Sugarbush North ski area in northern Vermont	696	<16	Unspecified	9 seasons (1972–1973/1980–1981)
Bøymo-Having et al. [28]	Dinghy sailing •	Swedish club sailors (Sweden)	21	Mean: 17 (SD: 1.30)	61.0	12 months
Dowling et al. [29]	Freestyle skiing	Participants in USSA (United States Ski Association) Freestyle competition (USA)	3180	10–20 (Mean 16)	73.0	4 seasons (1976–1977/1979–1980)
Ruedl et al. [30]	Alpine skiing ✭ & Snowboarding ✼	Participants to the first Winter Youth Olympic Games in Innsbruck in 2012 (Austria)	1021	14–18	56.3	10 days (2012)
	Sliding sports ✘				59.7	
					54.0	
Ruedl et al. [31]	Snowboarding	Participants to the 2015 Winter European Youth Olympic Festival (Austria and Liechtenstein)	899	14–18	59.4	5 days (2015)
Sran et al. [32]	Alpine skiing & Snowboarding	Students participating in a school program at a ski area in Alberta (Canada)	107	≤17	54.2	1 season (2013–2014)
Steffen et al. [33]	Alpine skiing, Freestyle skiing ⏣ & Snowboarding	Youth Olympic Winter Games Lillehammer 2016 (Norway)	1083	14–18	50.0	10 days (2016)
	Sliding sports ✘					10 days (2016)
	Skating sports ✦					10 days (2016)
Finch et al. [34]	Off-road motorcycle	Paediatric off-road motorcycle trauma attended by emergency medical services (EMS), Victoria (Australia)	1479	4–15	89.5	7 years (2010–2017)
Hagel et al. [35]	Alpine skiing & Snowboarding	Skiers and snowboarders in Quebec (Canada)	1,245,000	12–17	Unspecified	5 seasons (1995–2000)
Kim et al. [36]	Snowboarding	Injured snowboarders and skiers in a base-lodge clinic of a ski resort in Vermont (USA)	2260 ■	≤16	69.6	18 seasons (1988–2006)
	Alpine skiing		9465 ■		55.5	
Machold et al. [37]	Snowboarding	Students from 86 schools throughout (Austria)	2579	Mean: 14.7 (SD: 2.5)	68.0	1 week (season 1996/97)
Bergstrøm et al. [38]	Alpine skiing	Participants to the Alpine World Junior Championship, Bavallen, Voss (Norway)	998	15–19	54.7	6 days § ^ (1995)
Luo et al. [39]	Motocross	Motocross participants at a regional racetrack, Minnesota (USA)	202	5–17	91%	6 months (May-October 2010)

* recalculated from the data; • Classes: Europe & Laser; ✭ Disciplines: Alpine, Ski cross & Halfpipe; ✼Disciplines: Halfpipe & slopestyle; ✘ Disciplines: Bobsleigh, Luge & Skeleton; ✦ Disciplines: Figure, Short track & Speed; ⏣ Disciplines: Cross, Halfpipe & Slopestyle; §Info retrieved by: https://www.fis-ski.com; ■ Data including also those from adults.

**Table 2:** Outcomes reported in the 19 articles included in the systematic review.

Study, Year	Outcomes Reported				
	Concussion (N)	Denominator	Denominator Description	Incidence (95% CI)	Denominator (DAEs/PY) ^#^
Gil et al. [21]	2118	82,093,023 ✭	Population estimation (United States Census Bureau)	0.0259 (2.47 to 2.68)	PY
	1594	82,164,948 ✭		0.0194 (0.0184 to 0.184)	PY
Graves et al. [22]	39,364	162,661,000 ✭	Resort visits (National Ski Area Association)	0.242 (0.239 to 0.244)	DAEs
Emery et al. [23]	19	579 ✭	Questionnaire responders	0.032 (0.018 to 0.047)	PY
Macnab et al. [24]	54	816,837	Resort visits (ticket records)	0.07 (0.05 to 0.08)	DAEs
Macnab et al. [25]	19	142,098	Mountain’s lift (ticket records)	0.13 (0.07 to 0.19)	DAEs
Williams et al. [26]	8	204	Number of participants × riding days	39.2 (−27.78 to 106.2)	DAEs
Blitzer et al. [27]	43	138,132	Ticket records and comparison with a control population	0.31 (0.23 to 0.41)	DAEs
Bøymo-Having et al. [28]	7	2041.2	Estimation * (total reported hours of sailing: 5103 h)	3.43 (0.89 to 5.97)	DAEs
Dowling et al. [29]	7	10,188	Skier-days	0.69 (0.18 to 1.20)	DAEs
Ruedl et al. [30]	6	2220	Registered athletes in each sport × days of practice	2.70 (0.54 to 4.86)	DAEs
	5	1340		3.73 (0.47 to 7.00)	DAEs
Ruedl et al. [31]	3	395✭	Number of snowboarders × days of practice	7.59 (−0.96 to 16.15)	DAEs
Sran et al. [32]	17	16,260	Student-days	1.05 (0.55 to 1.54)	DAEs
Steffen et al. [33]	2	3170	Registered athletes in each sport × days of practice	0.63 (−0.24 to 1.51)	DAEs
	1	1390		0.72 (−0.81 to 2.25)	DAEs
	2	1660		1.20 (−0.46 to 2.87)	DAEs
Finch et al. [34]	82	6,662,162 ✭	Victorian population figures (Australian Bureau of Statistics 2016)	0.012 (0.009 to 0.14)	GP
Hagel et al. [35]	4101	8,925,000	Number of participants × days of practice (Canadian Ski Council)	0.46 (0.45 to 0.47)	DAEs
Kim et al. [36]	Unspecified	Unspecified	Lift ticket sales	0.0637 ✭	DAEs
	Unspecified	Unspecified		0.0277 ✭	DAEs
Machold et al. [37]	7	10,119	Snowboarder-days	0.69 (0.18 to1.20)	DAEs
Bergstrøm et al. [38]	1	5988	Participants × days of competitions	0.17 (−0.16 to 0.49)	DAEs
Luo et al. [39]	67	101	Number of participants/year	0.66 (0.57 to 0.75)	PY

^#^ Overall incidence rate calculated as: DAEs (concussions/1000 days of real exposure), PY (concussion/participant-year) GP (concussions/general popupulation of the considered area). ✭ Calculated from the available data. * Basing on personal experience in the same sailing class one of the Authors (FF) roughly estimated in 2.5 h the mean time of a sailing session.

**Table 3:** Study Quality.

First Author, Year	Cohort Selection	Analysis		Outcome Measurement					
	Representativeness of Exposed Cohort	Results Stratified by Important Factors (Sex/Age)	Difference in the Incidence Depending on Key Factors (Sex/Age Groups)	Outcome Ascertainment	Definitition of Concussion Provided	Mechanism of Injury	Study Follow-Up	Previous Hystory Reported	Practice or Competiton
Gil et al. [21]	Yes	Yes	Yes	Primary care provider or subspecialist	No	No	No	No	Unspecified
	Yes	Yes	Yes	Primary care provider or subspecialist	Yes	No	No	No	Unspecified
Graves et al. [22]	Yes	Yes	Yes	Physician	Yes	No	No	No	Unspecified
Emery et al. [23]	Yes	No	-	Physician, physiotherapist, athletic therapist, ambulance attendant, nurse)	Yes	No	No	No	Unspecified
Macnab et al. [24]	Yes	No	-	Physician	No	No	No	No	Unspecifid
Macnab et al. [25]	Yes	No	-	Physician	No	No	No	no	Unspecifid
Williams et al. [26]	Yes	No	-	Physician	No	No	No	No	Competition
Blitzer et al. [27]	Yes	Yes		Physician	No	No	No	No	Practice
Bøymo-Having et al. [28]	Yes	No	-	Physician	No	No	No	No	Training and competition
Dowling et al. [29]	Yes	No	-	Unspecified	No	No	No	No	Competition
Ruedl et al. [20]		No	-	Physician or physiotherapist	No	No	No	No	Competition
Ruedl et al. [31]	Yes	No	-	Physician or physiotherapist	No	No	No	No	Cometition
Sran et al. [32]	Yes	Yes	Yes	Sky patrol	No	No	No	No	Practice
Steffen et al. [33]		No	-	Medical staff	No	No	No	No	Competition
Finch et al. [34]	Yes	No	No	Staff of the emergency medical services	Yes	No	No	No	Leisure/Competition
Hagel et al. [35]	Yes	No	No	Ski patrollers	No	No	No	No	Unspecified
Kim et al. [36]	Yes	No	No	Unspecified	No	No	No	No	Unspecified
	Yes	No	No	Unspecified	No	No	No	No	Unspecified
Machold et al. [37]	Yes	No	No	Unspecified	No	No	No	No	Unspecified
Bergstrøm et al. [38]		Yes	Yes	Unspecified	No	Yes	No	No	Competition

## Supplementary Materials

The following are available online at https://www.mdpi.com/1660-4601/17/23/8728/s1.
