# Supplementary material for: The Incidence of Pediatric and Adolescent Concussion in Action Sports: A Systematic Review and Meta-Analysis
Source: Int J Environ Res Public Health. 2020 Nov 24;17(23):8728. doi: 10.3390/ijerph17238728 (PMC7727801; doi:10.3390/ijerph17238728)
Supplement: Supplementary file 1 [file ijerph-17-08728-s001.pdf]

Supplemantary material. Sample search strategy

Databases: PubMed and Web of Science.

(concussion or "brain concussion" or "brain injuries" or "mild traumatic brain injury" or "head injury" or injury) AND ("High-risk sport" or "Action sport" or "Extreme sport" or "Outdoor sport" or "Adventure sport" or Mountain-biking or "Mountain bike" or "cross country" or downhill or enduro or "Mountain bicycle" or "MTB" or "Freeride" or "trail" or "Slopestyle" or "Four-cross" or "Single-speed" or BMX or "Bicycle motocross" or X-games or motocross or motorcross or "dirt bike" or paragliding or "hang gliding" or "foot launched" or "speed flying" or "speed riding" or paramotoring or "powered paragliding" or parascending or parachuting or skydiving or "BASE jumping" or parachute or canopy or wingsuit or Climbing or "ice-climbing" or "rock climbing" or alpinism or bouldering or mountaineering or parkour or traceur or "skate boarding" or ″in-line skating″ or skateboarding or skateboard or Freestyle or longboarding or Windsurfing or boardsailing or sailboarding or Sailing or yachting or "kite surfing" or kitesurfing or kiteboarding or wakeboarding or "wake boarding" or "wave surfing" or surfing or "stand up paddle" or bodyboard or ski or skiing or snowboard or snowboarding or snowsport or "snow sports" or snowkiting) AND (Adolescent or Youth or Child or School or Students) AND ("Cross-Sectional Studies" or "Prevalence" or "Cohort Studies" or "Follow-up Studies" or "Incidence")
